# Influence of past advanced behavior guidance experience on parental acceptance for autistic individuals in the dental setting

**DOI:** 10.1186/s12903-023-02716-6

**Published:** 2023-01-17

**Authors:** Apichaya Manopetchkasem, Natchalee Srimaneekarn, Pattarawadee Leelataweewud, Apiwan Smutkeeree

**Affiliations:** 1 Department of Pediatric Dentistry, Faculty of Dentistry, Mahidol University, Bangkok, 10400 Thailand; 2 Department of Anatomy, Faculty of Dentistry, Mahidol University, Bangkok, 10400 Thailand

**Keywords:** Autism, Behavior guidance techniques, Parental acceptance, Passive restraint, Pharmacological management

## Abstract

**Background:**

Autism is a lifelong neurodevelopmental disorder that poses challenges during dental treatment. Advanced behavior guidance techniques (BGTs) have been used to provide dental care for autistic people who have specific characteristics and complex dental treatment. This study was conducted to evaluate parental acceptance and analyze parents’ opinions of advanced BGTs during dental treatment in autistic people.

**Methods:**

This cross-sectional study was conducted on 141 parents of autistic people from the Mahidol Dental Hospital and the Autism online community. Informed consent was obtained before enrolling participants in the study. All parents were asked to rate their acceptance after watching VDO clips: passive restraint by device (PRBD), oral sedation (OS), and general anesthesia (GA) to evaluate parental acceptance of advanced BGTs through an online questionnaire survey. The online questionnaire included a visual analog scale (VAS) and open-ended questions to collect their opinions on each advanced BGT. Participants were categorized into two subgroups as follows: 81 in the “Experience group” and 60 in the “No experience group” according to their autistic people’ advanced BGT experience. Friedman’s two-way analysis of variance and the Mann–Whitney U test were used for statistical analyses. Open-ended questions were analyzed using quantitative content analysis.

**Results:**

PRBD was ranked the highest, followed by GA and OS. Parents in the “Experience group” rated significantly higher acceptance of their BGT experience than parents in the “No experience group” in all the three advanced BGTs.

**Conclusions:**

All advanced BGTs were particularly accepted in this study. Previous experience of advanced BGTs had an influence on parental acceptance. Parents commented on their opinions toward each advanced BGT with a variety of perspectives.

*Trial registration*: The protocol was approved by the ethical committee of the Faculty of Dentistry/Faculty of Pharmacy, Mahidol University (COA.No.MU-DT/PY-IRB 2021/022.1702) and was registered with Thai Clinical Trials Registry (TCTR20220521001).

## Background

Autism is a neurodevelopmental disorder that typically appears during the first 3 years of life and is characterized by specific autistic experiences and characteristics including specialized, focused, or intense interests [1, 2]. People diagnosed with autism are more likely to experience difficulties in activities of daily living and are more likely to report challenges regarding access to dental healthcare [3–9]. Autistic people show a higher frequency and more serious periodontal problems due to poor oral hygiene and inability to clean. Nevertheless, data on the prevalence of dental caries in these people are still controversial, with some studies reporting low caries prevalence but others reporting high prevalence compared with non-autistic people [10–12].

In 2019, 80% of autistic adults reported difficulty visiting a general practitioner [9]. Only 56% of autistic people in Thailand can access healthcare services due to their specific autistic characteristics [13]. The American Academy of Pediatric Dentistry (AAPD) published a guideline on behavior guidance techniques (BGTs) in 2020 for pediatric patients with dental problems, including those with special health care needs (SHCN). AAPD classified BGTs into two groups: basic BGTs and advanced BGTs. The basic BGTs consist of communication guidance, positive pre-visit imagery, direct observation, tell-show-do, ask-tell-ask, voice control, nonverbal communication, positive reinforcement and descriptive phase, distraction, memory restructuring, parental presence/absence, and nitrous oxide/oxygen inhalation. Furthermore, the advanced BGTs include protective stabilization, sedation, and general anesthesia. [14]

Autistic people have specific behaviors and require different BGTs from non-autistic people [3, 5, 7, 10, 11]. However, there are no specific recommendations or BGT guidelines for autistic people in dental treatment [14]. Most of the basic BGTs require communication between the dentist and the patient; therefore, some autistic individuals may not be managed by basic BGTs [3, 15–17]. Advanced BGTs, such as protective stabilization, oral sedation (OS), and general anesthesia (GA), have been proposed to manage autistic individuals [14]. However, not all advanced BGTs can be applied to everyone, and not all advanced BGTs will be accepted by the parents [14, 18].

A few studies have evaluated parental acceptance of advanced BGTs in SHCN patients. Previous studies published from 1995 to 2013 reported that basic BGTs received higher acceptance rates than advanced BGTs [19, 20]. Furthermore, the parental acceptance of advanced BGTs for SHCN patients was higher than that for healthy children [19, 21]. In a study on the parents of autistic people conducted by Marshall in 2008 [20], the acceptance rates of GA and active restraint by parents were found to be > 90%. Advanced BGTs, such as active restraint by staff and passive restraint by device (PRBD), had lower acceptance rates than other BGTs.

In recent years, parents’ perceptions of behavioral management techniques in dental treatment have changed and continue to change over time [22–25]. Even though BGTs have not changed much over time, dynamic changes, such as changes in parenting styles and the internet and social media, might affect parental acceptance of BGTs and their perspective on health care. To our knowledge, no study has focused on parental acceptance of advanced BGTs in autistic people. Therefore, the present study was performed to compare parental acceptance of advanced BGTs, such as PRBD, OS, and GA, between autistic people who have and have not experienced advanced BGTs in a dental setting and to analyze parents’ opinions of advanced BGTs. The findings of this study can be helpful for dental practitioners in parental explanation, discussion, and providing BGTs to deliver successful and satisfactory dental treatment for autistic individuals. The null hypothesis of this study was that there would be no differences in the level of parental acceptance toward passive restraint by device and pharmacological management in the dental setting between parents of autistic people with prior advanced BGT experience and parents of those without BGT experience.

## Methods

This cross-sectional study was approved by the ethical committee of the Faculty of Dentistry/Faculty of Pharmacy, Mahidol University in accordance with the ethical standards laid down in the 1964 World Medical Association Declaration of Helsinki and its later amendments and was registered with Thai Clinical Trials Registry. Based on the results of Marshall et al. [20] and a two-tailed test, a sample size of 53 parents of autistic people with advanced BGT experience and those without any BGT experience per group was required for this study. Due to the COVID-19 pandemic in Thailand, data collection was performed via online questionnaires, The previous online questionnaire study had a response rate of approximately 55%. To compensate for the non-responders, the number of participants in this study was increased to 100 parents per group for a total of 200 parents.

Parents (father or mother) of autistic people, aged < 35 years, from Mahidol University dental hospital and members of the Autism online community, which consisted of Thai parents of autistic people, were invited to participate in this study. All participants were contacted individually via phone calls and briefed about the research objective and the procedures of the study by a single operator. Then, they were allowed to ask questions about this study if any. Subsequently, informed consent was obtained from all parents before enrolling them in the study. Parents who were blind, deaf, had language difficulties, or unable to read and understand Thai language were excluded.

The online questionnaire survey was conducted via Surveymonkey.com (Survey Monkey Inc., California, USA) and was divided into two primary components. The first component was designed to collect data concerning the parents and their autistic children, including their advanced BGT experience. Parents were divided into two subgroups, viz., the “Experience group” and the “No experience group,” according to their child’s advanced BGT experience (PRBD, OS, or GA) based on the AAPD behavior guidance for pediatric dental patients published in 2020 [14]. This study focused only on PRBD, which was previously reported to have low parental acceptance [20, 22–25]. Pharmacological management, such as OS and GA, is commonly used in pediatric dentistry. Therefore, the second component of the questionnaire was designed to collect data regarding parental acceptance of the three advanced BGTs after watching a video clip, including PRBD, OS, and GA, using a visual analog scale (VAS) on a sliding scale, where the scores can be automatically converted into integer numbers. The VAS scores ranged from 0 to 100, representing “Totally disagree” to “Totally agree,” respectively. Parents can type their opinions toward each BGT in a comment box responding to an open-ended question composed of questions about their opinions on each BGT technique, including positive and negative feedback. The BGTs video clips included role-playing by a dentist, dental assistants, staff, and a simulated patient. The total length of BGT video clips did not exceed 1 min for each technique. There were Thai subtitles for the BGT names, and Thai audio described each BGT in a short explanation along with video clips. The sequence of the BGT video clips was randomized into six sets to minimize study bias.

The validity of the questionnaire was evaluated using the index of item-object congruence (IOC) from three pediatric dentists. The IOC for each question had a score of 0.67–1.00. The reliability of the questionnaire was checked by 10 parents who were invited to complete the questionnaire and were retested after a month. The intraclass correlation coefficient values ranged from 0.8 to 1.0.

Parents were required to complete both component 1 and component 2 before submitting the online questionnaire. After watching each random advanced BGT video clip, they had to rate the VAS to represent their level of acceptance within 15 s and had 3 min to comment on each BGT after watching each technique before moving to the next technique. Then, the parents continued performing the same steps until they completed all three advanced BGTs.

### Statistical analysis

Data were analyzed using the Statistical Package for the Social Sciences (SPSS Inc., IBM, Chicago, IL, USA) for Mac version 25.0. The level of confidence was set at 95%, and a p value of < 0.05 was considered to be statistically significant. Mean VAS scores were used to rank the rate of acceptance of each BGT. Friedman’s two-way analysis of variance was used to analyze the difference in VAS scores among the advanced BGTs. The Mann–Whitney U test was conducted to compare VAS scores among the advanced BGTs between the “Experience group” and the “No experience group.” Open-ended questions were analyzed using quantitative content analysis. All open-ended answers were coded and categorized by two investigators (AM and NS) as positive and negative feedback. Any disagreement between two investigators was resolved through a decision by the third investigator (AS). Descriptive statistics were used to analyze parents’ opinions toward each advanced BGT.

## Results

A total of 141 parents of autistic people consented and completed both components of the online questionnaire. In total, 81 parents whose children had experienced at least one advanced BGT (PRBD, OS, or GA) were categorized into the “Experience group,” and the remaining 60 parents whose children had not experienced any advanced BGT were assigned into the “No experience group.”

Most parents were mothers (79.4%), 56% of parents were generation X or those born between 1965 and 1980 [26], 51.1% of parents had a bachelor’s degree, and 51.8% had low family income (less than 500 USD per month). There was no significant difference in the demographic data of the “Experience group” and the “No experience group.” as shown in Table 1.

**Table 1:** Demographic data and socio-environment of parents

Demographic	Total (%) (N = 141)	Advanced BGT		*p* value
		Exp (%) (N = 81)	No exp (%) (N = 60)	
*Parent’s*				
*Age*				0.270
Gen Y (25–40 y)	39 (27.7)	19 (23.5)	20 (33.3)	
Gen X (41–56 y)	79 (56.0)	46 (56.8)	33 (55.0)	
Baby boomer (> 56 y)	23 (16.3)	16 (19.8)	7 (11.7)	
*Gender*				0.654
Male	21 (14.9)	13 (16.0)	8 (13.3)	
Female	120 (85.1)	68 (84.0)	52 (86.7)	
*Relation to autistic people*				0.650
Mother	112 (79.4)	65 (80.2)	47 (78.3)	
Father	16 (11.3)	10 (12.3)	6 (10.0)	
Other	13 (9.2)	6 (7.4)	7 (11.7)	
*Education*				0.570
Primary school	8 (5.7)	4 (4.9)	4 (6.7)	
High school	30 (21.3)	16 (19.8)	14 (23.3)	
Diploma	18 (12.8)	8 (9.9)	10 (16.7)	
Bachelor’s	72 (51.1)	46 (56.8)	26 (43.3)	
Master’s or higher	13 (9.2)	7 (8.6)	6 (10.0)	
*Income (per month)*				0.368
< 500 USD	73 (51.8)	43 (53.1)	30 (50.0)	
500–< 800 USD	25 (17.7)	12 (14.8)	13 (21.7)	
800–< 1300 USD	21 (14.9)	15 (18.5)	6 (10.0)	
> 1300 USD	22 (15.6)	11 (13.6)	11 (18.3)	

*Exp*  Advanced BGT experience, *No exp*  No advanced BGT experience

The mean age of autistic people was 14.43 years (range: 3–34 years). The majority of them were male (77.3%) and had various autistic support needs/levels (level 1 = 31.2%, level 2 = 35.5%, and level 3 = 33.3%). Furthermore, all autistic people in the “Experience group” had received dental treatment, with the proportion being statistically significantly different from that in the “No experience group” where some of them had received no previous dental treatment (Table 2).

**Table 2:** Demographic data of autistic people

Demographic	Total (%) (N = 141)	Advanced BGT		*p* value
		Exp (%) (N = 81)	No exp (%) (N = 60)	
*Autistic people’s*				
*Age*				0.942
Preschool (3–5 y)	14 (9.9)	7 (8.6)	7 (11.7)	
School age (6–12 y)	55 (39.0)	32 (39.5)	23 (38.3)	
Adolescent (13–18 y)	37 (26.2)	22 (27.2)	15 (25.0)	
Adult (19–35 y)	35 (24.8)	20 (24.7)	15 (25.0)	
*Gender*				0.511
Male	109 (77.3)	61 (75.3)	48 (80.0)	
Female	32 (22.7)	20 (24.7)	12 (20.0)	
*Autistic support needs/levels*				0.415
Level 1 “Requiring support”	44 (31.2)	27 (33.3)	17 (28.3)	
Level 2 “Requiring substantial support”	50 (35.5)	25 (30.9)	25 (41.7)	
Level 3 “Requiring very substantial support”	47 (33.3)	29 (35.8)	18 (30.0)	
*Past dental experience*				0.000*
Yes	127 (90.1)	81 (100.0)	46 (76.7)	
No	14 (9.9)	0	14 (23.3)	
*BGTs experience*				
PRBD	69 (48.9)	69 (85.2)	0	
OS	8 (5.7)	8 (9.9)	0	
GA	39 (27.7)	39 (48.1)	0	

*Exp*  Advanced BGT experience, *No exp*  No advanced BGT experience, statistically significant difference (*p < 0.01)

### Parental acceptance of advanced BGTs

The mean VAS score was ranked from the highest to the lowest, respectively, for PRBD (VAS = 76.3), GA (VAS = 69), and OS (VAS = 68), and all BGTs achieved a mean VAS score of > 68, which was considered to be acceptable [27]. PRBD was the only BGT that had a significantly higher parental acceptance rate than OS and GA (*p* < 0.05), as shown in Table 3.

**Table 3:** Parental acceptance of each advanced BGT in parents of autistic people

BGTs	VAS Means ± SD	VAS Median (IQR)	VAS Range
PRBD	76.3 ± 30.2	90.0 (39)^a^	0–100
OS	68.0 ± 28.7	70.0 (50)^b^	0–100
GA	69.0 ± 32.3	80.0 (50)^b^	0–100

The different superscript letters indicate significant differences among BGTs

Related-samples Friedman’s two-way analysis of variance by ranks, statistically significant difference (*p* < 0.05)

*PRBD*  Passive restraint by device, *OS*  Oral sedation, *GA*  General anesthesia

### Comparison of parental acceptance of advanced BGTs between the experience and no experience groups

Table 4 shows the level of parental acceptance of advanced BGTs between the experience and no experience groups. The ranking of parental acceptance of the advanced BGTs in the “Experience group” from the highest to the lowest was in the order of PRBD (VAS = 99), GA (VAS = 76), and OS (VAS = 70). The “No experience group” also showed the highest acceptance ranking for PRBD and GA (VAS = 80), followed by OS (VAS = 68.5). However, only the PRBD parental acceptance level was significantly different between the two groups (*p* = 0.036), where PRBD acceptance was rated higher by the “Experience group” than the “No experience group.”

**Table 4:** Parental acceptance of advanced BGTs in the experience and no experience groups

Advanced BGT experience	At least one BGT experience VAS Median (IQR)			PRBD experience VAS Median (IQR)			OS experience VAS Median (IQR)			GA experience VAS Median (IQR)		
BGTs	Exp (n = 81)	No exp (n = 60)	*p* value	Exp (n = 69)	No exp (n = 72)	*p* value	Exp (n = 8)	No exp (n = 133)	*p* value	Exp (n = 39)	No exp (n = 102)	*p* value
PRBD	99.0 (31)	80.0 (48)	0.036*	100.0 (30)	80.0 (50)	0.009*	100.0 (0)	86.0 (48)	0.002*	90.0 (50)	90.0 (31)	0.569
OS	70.0 (50)	68.5 (50)	0.981	66.0 (46)	70.0 (50)	0.232	100.0 (14)	67.0 (50)	0.007*	70.0 (50)	66.5 (50)	0.294
GA	76.0 (50)	80.0 (53)	0.391	68.0 (50)	84.0 (40)	0.018*	100.0 (0)	78.0 (50)	0.002*	91.0 (35)	76.5 (60)	0.033*

Independent-samples, Mann–Whitney U test, statistically significant difference (*p < 0.05)

*Exp*  Advanced BGT experience, *No exp*  No advanced BGT experience,

*PRBD*  Passive restraint by device, *OS*  Oral sedation, *GA*  General anesthesia

Regarding the experience of each advanced BGT, the parents of autistic individuals with PRBD experience rated significantly high PRBD acceptance (*p* = 0.009), but they rated significantly low GA acceptance (*p* = 0.018). Similarly, the parents of autistic individuals with previous GA experience rated significantly higher GA acceptance than those without GA experience (*p* = 0.033). In contrast, the parents of autistic individuals with previous OS experience rated significantly higher acceptance for all three advanced BGTs than those of autistic individuals who never had OS experience (*p* < 0.05), as shown in Table 4.

### Open-ended questions

In total, 139 (98.6%) parents provided their opinions about each advanced BGT, including PRBD, OS, and GA. They answered the open-ended questions with a variety of perspectives, but their opinions could be grouped into positive and negative. Interestingly, several parents commented that they intended to use advanced BGTs only when necessary or according to the dentist’s recommendation for all types of advanced BGTs.

#### Passive restraint by device

There were 69 parents in the “PRBD experience group” and 70 parents in the “No PRBD experience group.” The parents in both groups trusted that PRBD use can reduce autistic individuals’ movements and help the dentist achieve successful and safe treatment. However, 29.5% of parents believed that PRBD use can cause fear during treatment, which could contribute to future uncooperative behavior. Some parents were not confident about its effectiveness when used for autistic individuals who were mature or very strong and believed that it could cause physical trauma to their child. As shown in Table 5, parents in both groups had similar positive opinions, but a higher number of parents in the “PRBD experience group” expressed positive opinions on “Achieve treatment” than parents in the “PRBD no experience group.” In addition, parents in the “PRBD no experience group” had more negative opinions than parents in the “PRBD experience group.”

**Table 5:** Parents’ opinions toward PRBD used for their autistic children

PRBD	N = 139 No. (%)	PRBD experience			
		Exp (n = 69)		No exp (n = 70)	
		No. (%)	Median (IQR)	No. (%)	Median (IQR)
*Positive opinions*					
(+) Reduce movement	38 (27.3)	20 (29.0)	100.0 (14.8)	18 (25.7)	89.5 (20.0)
(+) Achieve treatment	31 (22.3)	19 (27.5)	70.0 (50.0)	12 (17.1)	83.0 (20.0)
(+) Safe	31 (22.3)	16 (23.2)	100.0 (10.0)	15 (21.4)	91.0 (30.0)
(+) Easy to use	2 (1.4)	1 (1.4)	100.0 (0.0)	1 (1.4)	80.0 (0.0)
*Negative opinions*					
(−) Fearful	41 (29.5)	19 (27.5)	70.0 (60.0)	22 (31.4)	70.0 (55.8)
(−) Physical trauma	18 (12.9)	7 (10.1)	70.0 (30.0)	11 (15.7)	78.0 (28.0)
(−) Unreliable outcome	13 (9.4)	6 (8.7)	99.5 (24.5)	7 (10.0)	80.0 (50.0)
(−) Enforcement	13 (9.4)	5 (7.2)	92.0 (31.0)	8 (11.4)	27.0 (37.3)

*Exp*  Advanced BGT experience, *No exp*  No advanced BGT experience, *PRBD*  Passive restraint by device, +  = Positive opinion, −  Negative opinion

#### Oral sedation

Only eight autistic individuals in this study had OS experience. Approximately 37.4% of parents in both groups believed that OS caused calmness in autistic individuals and helped them undergo dental treatment (15.1%). Moreover, some parents believed that OS was safe, no restraint was needed, there was no pain, and it still maintained their consciousness. In contrast, 34.5% of parents were worried about the side effects of drugs used. Other negative opinions included unreliable outcomes, unfamiliar techniques, and autistic individual’s consciousness during OS procedures that may have caused negative experiences in terms of being conscious to their children (Table 6). When compared between groups, most parents in the “OS experience group” but only 35.1% in the “OS no experience group” believed that OS could calm down autistic individuals. The only negative opinion from parents in the “OS experience group” was the side effects of OS; however, parents in the “OS no experience group” commented on other negative opinions, as shown in Table 6.

**Table 6:** Parents’ opinions toward OS used for their autistic children

OS	N = 139 No. (%)	OS experience			
		Exp (n = 8)		No exp (n = 131)	
		No. (%)	Median (IQR)	No. (%)	Median (IQR)
*Positive opinions*					
(+) Calm down	52 (37.4)	6 (75.0)	100.0 (15.0)	46 (35.1)	70.0 (47.8)
(+) Achieve treatment	21 (15.1)	1 (12.5)	90.0 (−)	20 (15.3)	68.0 (44.0)
(+) Being conscious	8 (5.8)	0 (0.0)	−	8 (6.1)	66.5 (45.0)
(+) No restraint	7 (5.0)	1 (12.5)	100.0 (−)	6 (4.6)	100.0 (34.0)
(+) Safe	6 (4.3)	0 (0.0)	−	6 (4.6)	70.5 (37.5)
(+) No pain	2 (1.4)	0 (0.0)	−	2 (1.5)	85.0 (−)
*Negative opinions*					
(−) Side effects	48 (34.5)	2 (25.0)	80.0 (−)	46 (35.1)	50.0 (30.0)
(−) Unreliable outcome	14 (10.1)	0 (0.0)	−	14 (10.7)	65.0 (36.0)
(−) Unfamiliar technique	9 (6.5)	0 (0.0)	−	9 (6.9)	40.0 (31.0)
(−) Being conscious	5 (3.6)	0 (0.0)	−	5 (3.8)	56.0 (66.5)

*Exp*  Advanced BGT experience, *No exp*  No advanced BGT experience, *OS*  Oral sedation,

+  = Positive opinion, − = Negative opinion

#### General anesthesia

Approximately 40.3% of parents trusted that GA use can help undergo dental treatment, especially for autistic individuals who had uncooperative behavior or required extensive dental care. Some parents gave positive opinions such as no psychological trauma, no pain, safe, and no restraint required. Nevertheless, 65% of parents were worried about the side effects of GA. A controversy concerning their unconsciousness was observed, wherein some parents reported that GA was good because autistic individuals cannot resist, whereas some parents were worried about their unconsciousness during GA procedures. Other negative opinions included time-consuming procedure and cause of fear in some autistic individuals, as shown in Table 7. Parents in the “GA experience group” provided more positive opinions than parents in the “GA no experience group,” especially on “Achieve treatment.” Only unconsciousness status caused significantly more worry for parents in the “GA no experience group” than for parents in the “GA experience group.”

**Table 7:** Parents’ opinions toward GA used for their autistic children

GA	N = 139 No. (%)	GA experience			
		Exp (n = 39)		No exp (n = 100)	
		No. (%)	Median (IQR)	No. (%)	Median (IQR)
*Positive opinions*					
( +) Achieve treatment	56 (40.3)	20 (51.3)	82.0 (33.0)	36 (36.0)	80.0 (35.0)
( +) No resistance	26 (18.7)	9 (23.1)	91.0 (24.5)	17 (17.0)	74.0 (29.5)
( +) No psychological trauma	13 (9.4)	6 (15.4)	96.6 (38.8)	7 (7.0)	80.0 (28.0)
( +) No pain	11 (7.9)	4 (10.3)	95.0 (17.0)	7 (7.0)	75.0 (50.0)
( +) Safe	10 (7.2)	4 (10.3)	90.0 (31.3)	6 (6.0)	100.0 (25.0)
( +) No restraint	7 (5.0)	2 (5.1)	85.0 (−)	5 (5.0)	100.0 (38.0)
*Negative opinions*					
(−) Side effects	91 (65.5)	15 (38.5)	74.0 (34.0)	41 (41.0)	50.0 (69.0)
(−) Being unconscious	20 (14.4)	1 (2.6)	76.0 (−)	19 (19.0)	15.0 (74.5)
(−) Time−consuming	2 (1.4)	2 (5.1)	64.5 (−)	0 (0.0)	−
(−) Terrifying	2 (1.4)	1 (2.6)	50.0 (−)	1 (1.0)	50.0 (−)

*Exp*  Advanced BGT experience, *No exp*  No advanced BGT experience,

*GA*  General anesthesia, +  = Positive opinion, − = Negative opinion

## Discussion

Autistic people have limitations during dental treatment due to their specific autistic experiences and characteristics and specialized, focused, or intense interests, which require more advanced behavior management techniques [1–3, 14]. Advanced BGTs based on physical restraint and pharmacological usage may be considered an invasive procedure by some parents. In this study, three advanced BGTs, PRBD, OS, and GA, were included because several previous studies have reported low parental acceptance rates from parents of both healthy children and SHCN patients in several countries [19, 23, 24, 27]. A VAS was used to evaluate parental acceptance of behavior management techniques. The VAS scores ranged from 0 to 100, representing “Totally disagree” to “Totally agree,” and the mid-point (score of 50) indicated neutrality or ambivalence to the technique. VAS scores above or below 50 represented acceptable or not-acceptable responses from parents [27]. The results of this study showed that parents of autistic people rated the three advanced BGTs with mean VAS scores of > 68, indicating that it was acceptable. PRBD gained the highest acceptance rate, followed by GA and OS. These results are consistent with those of previous studies on the parents of healthy children in Thailand [28] and on Brazilian parents of patients with SHCNs [19, 21] who accepted PRBD over other pharmacological management techniques. However, some studies in the USA, Germany, and Middle East countries reported that parents of both healthy children and patients with SHCNs including autistic people rated PRBD the lowest [20, 22–25, 27]. To our knowledge, this is the first study to compare parental acceptance of the experience of advanced BGTs between autistic people with and without advanced BGTs experience. It was observed that the parents of autistic people who had experience of at least one advanced BGT (PRBD, OS, or GA) reported significantly higher acceptance for PRBD than the parents of those with no experience of advanced BGTs. This finding could be related to a significant association between previous advanced BGT experience and parental acceptance of that technique. Furthermore, almost half of the autistic people in this study had PRBD experience, which was higher than the proportion of autistic people who had GA and OS experience.

Parents in this study reported three autistic support needs/levels for their children based on the description of specific autistic experiences and characteristics, including specialized, focused, or intense interests that were provided in the questionnaire [1]. Parents of autistic level 3 individuals “Requiring very substantial support” rated their acceptance towards PRBD significantly more than parents of children with autistic level 2 or “Requiring substantial support” (Table 8). This difference in ranking may be due to uncontrolled behavior of the autistic individuals, requiring physical restraint to prevent any accident during dental treatment.

**Table 8:** Parental acceptance of advanced BGTs among different autistic support needs/level

BGTs	Autistic level [Median VAS (IQR)]			
	Level 1 (n = 44)	Level 2 (n = 50)	Level 3 (n = 47)	*p* value
PRBD	85.0 (50)	80.0 (52)	100.0 (20)	0.001*
OS	65.5 (41)	70.0 (46)	70.0 (56)	0.639
GA	77.0 (44)	80.0 (52)	80.0 (58)	0.872

Independent-Samples, Kruskal–Wallis test, statistically significant difference (*p < 0.05)

*PRBD*  Passive restraint by device, *OS*  Oral sedation, *GA*  General anesthesia

PRBD was the most common advanced BGT for patients with uncooperative behavior, and almost half of the autistic individuals in this study had a previous experience. The open-ended parental opinions showed that parents in the “PRBD no experience group” tended to express more negative opinions and less positive opinions than parents in the “PRBD experience group.” Parents in the “PRBD experience group” believed that PRBD usage was sufficient to reduce autistic individuals’ movements and achieve safe and successful dental treatment. However, some parents in both groups believed PRBD was the BGT that forced their autistic children and preferred to use PRBD only when necessary or according to the dentist’s recommendation.

OS was the least common advanced BGT for autistic individuals in Thailand. The parents of autistic individuals who had OS experience reported significantly higher acceptance of OS than the parents of autistic individuals who had no OS experience. This finding correlated with those of previous studies on healthy children and autistic individuals [20, 22, 28, 29]. Interestingly, a previous OS experience of their child can increase parental acceptance of all three advanced BGTs, i.e., PRBD, OS, and GA. This may be because the OS procedure contains physical restraint by a device and sedative drug consumption, which is similar to PRBD and GA procedures. However, only 8 (5.7%) autistic individuals had OS experience in this study; hence, this finding must be confirmed by further studies. The open-ended parental opinions in the “OS experience group” showed that parents in both groups believed that OS could calm their child and help the child undergo the dental treatment, but they were also worried about the side effects of drug use. Parents of autistic individuals who had never experienced OS also reported other negative opinions, for instance, unreliable outcome and unfamiliar techniques. Moreover, OS procedures still maintain autistic individuals’ consciousness that received both positive and negative feedback from parents. Therefore, it is necessary to discuss the explanation about OS procedures, including parental expectation, before using OS in dental treatment for autistic individuals.

GA had been the most unaccepted advanced BGT for children in the dental setting [19, 21, 27, 30]. However, it has currently gained more parental acceptance over time because of trustworthy effectiveness and decreased tendency of side effects. This study showed that GA acceptance was significantly less than PRBD acceptance, which is not consistent with some previous studies that reported that parental acceptance of GA was higher than that of PRBD [22–25]. In Thailand, the dental management of autistic individuals having cooperation problems tends to require comprehensive oral rehabilitation under pharmacological management to provide good-quality dental care. Nevertheless, a number of parents cannot afford the cost of dental treatment under pharmacological management or are uncomfortable with the pharmacological management procedures and related complications and prefer comprehensive dental treatment with the use of PRBD instead. Moreover, dental treatment under GA can only be performed in the tertiary care dental hospital with a long waiting queue. These factors may also be responsible for the parental acceptance of PRBD over GA. The parents of autistic individuals who had GA experience reported significantly higher GA acceptance than the parents of autistic individuals who had no GA experience. This finding reinforced the results of previous studies on healthy children and autistic individuals [20, 22, 28].

In the open-ended questions, the majority of parents answered that they were worried about the side effects of several drugs used in GA. Parents in the “GA experience group” commented that their child can undergo dental treatment without developing psychological trauma. However, there were no differences in negative opinions between both groups, except for autistic individuals’ unconsciousness. Parents in the “GA no experience group” were worried about unconsciousness condition, whereas those in the “GA experience group” believed that unconsciousness was an advantage to undergo dental treatment. Finally, parents in both groups intended to use GA for their autistic children only when necessary or according to the dentist’s recommendation.

Regarding the limitations of this study, the questionnaire used in this study was modified to an online questionnaire because of the COVID-19 pandemic situation. The questionnaire used the sliding scale instead of value collections, and hence the data had to be converted into integer numbers and not decimal numbers. Nevertheless, previous research has mentioned that BGT explanation details and video presentation sequences can impact parental acceptance [22]. The sequence of BGT video clips in this study was randomized and provided only a brief description of each BGT. Furthermore, this study did not clarify the dental procedures, which may affect parental acceptance of BGT selection; for instance, parents may accept advanced BGTs more when their child requires an urgent or complex dental treatment than when their child requires a routine dental check-up.

Based on the study results, parents tended to accept all three advanced BGTs for their autistic children in dental practice; however, several parents expressed concerns such as the side effects of pharmacological management, physical and psychological trauma, and even doubts regarding BGT effectiveness. Dentists must discuss the indications of use and risks and benefits with parents when they consider using advanced BGTs for autistic individuals. This approach can contribute toward achieving successful and satisfactory dental treatment for parents, patients, and dentists.

## Conclusion

Parental acceptance of advanced BGTs in autistic individuals during dental treatment was ranked in the order of PRBD, GA, and OS. All three advanced BGTs were particularly accepted, and parents intended to use advanced BGTs only when necessary or according to the dentist’s recommendation.

## Data Availability

The datasets used and analyzed during the current study are available from the corresponding author upon reasonable request.

## Abbreviations

AAPD: American Academy of Pediatric Dentistry
BGT: Behavior guidance techniques
GA: General anesthesia
ICC: Intraclass correlation coefficient
IOC: Item-object congruence
OS: Oral sedation
PRBD: Passive restraint by device
SHCN: Special health care needs
SPSS: Statistical Package for the Social Sciences
VAS: Visual analog scale
